# Aggregation tendency of guest Fe in NaCo_1−*x*_Fe_*x*_O_2_ (*x* < 0.1) as investigated by systematic EXAFS analysis

**DOI:** 10.1038/s41598-020-68147-3

**Published:** 2020-07-09

**Authors:** Toshiaki Moriya, Hideharu Niwa, Hiroaki Nitani, Yutaka Moritomo

**Affiliations:** 1 Graduate School of Pure and Applied Sciences, University of Tsukuba, Tsukuba, 305-8571 Japan; 2 Faculty of Pure and Applied Sciences, University of Tsukuba, Tsukuba, 305-8571 Japan; 3 Tsukuba Research Center for Energy Materials Science (TREMS), University of Tsukuba, Tsukuba, 305-8571 Japan; 4 Institute of Materials Science, High Energy Accelerator Research Organization (KEK), Tsukuba, 305-0801 Japan

**Keywords:** Characterization and analytical techniques, Structure of solids and liquids, Materials science, Materials for energy and catalysis, Batteries

## Abstract

In transition metal (*M*) compounds, the partial substitution of the host transition metal (*M*_h_) to guest one (*M*_g_) is effective to improve the functionality. To microscopically comprehend the substitution effect, degree of distribution of *M*_g_ is crucial. Here, we propose that a systematic EXAFS analysis against the *M*_g_ concentration can reveal the spatial distribution of *M*_g_. We chose NaCo_1−*x*_Fe_*x*_O_2_ as a prototypical *M* compound and investigated the local intermetal distance around the guest Fe [*d*_Fe–*M*_(*x*)] against Fe concentration (*x*). *d*_Fe–*M*_(*x*) steeply increased with *x*, reflecting the larger ionic radius of high-spin Fe^3+^. The *x*-dependence of *d*_Fe–*M*_(*x*) was analyzed by an empirical equation, ${d}_{\mathrm{F}\mathrm{e}-M}(x)=sxd_{\mathrm{F}\mathrm{e}-\mathrm{F}\mathrm{e}}+(1-sx)d_{\mathrm{F}\mathrm{e}-\mathrm{C}\mathrm{o}}$, where *d*_Fe–Fe_ and *d*_Fe–Co_ are the Fe–Fe and Co–Fe distances, respectively. The parameter *s* represents degree of distribution of Fe; *s* = 1, > 1, < 1 are for random, attractive, and repulsive distribution, respectively. The obtained *s* value (= 4.8) indicates aggregation tendency of guest Fe.

## Introduction

**Figure 1:**
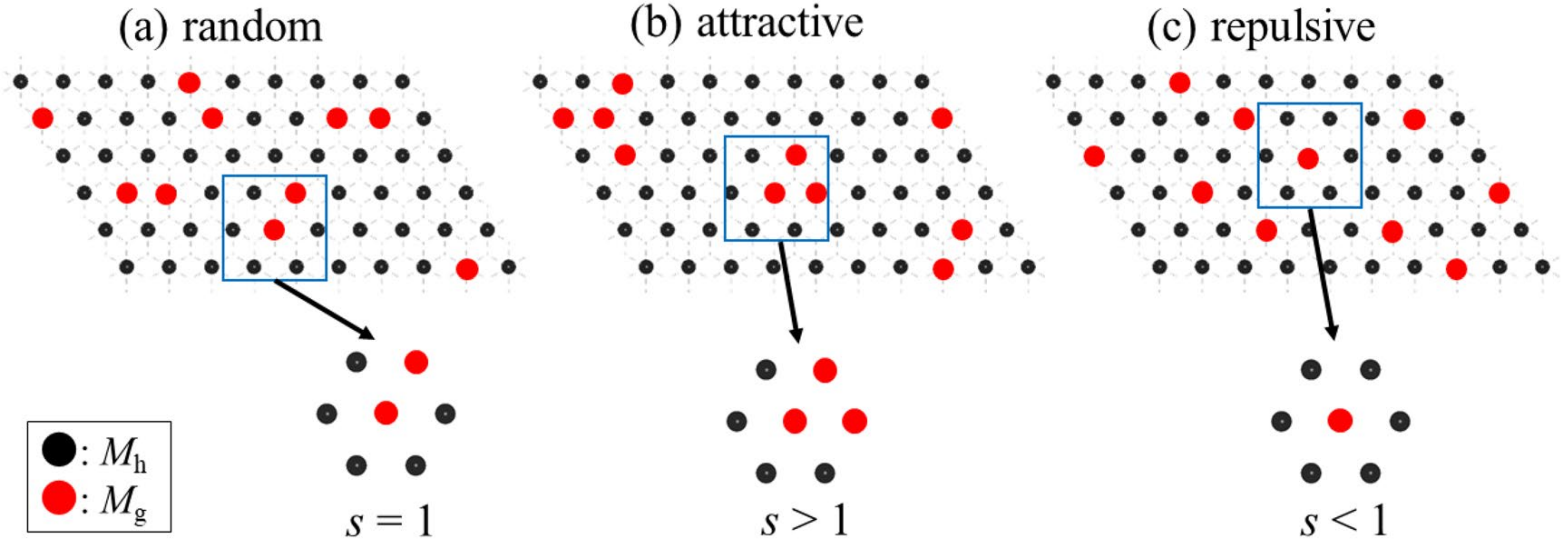
Distribution of the partial substituted element (*M*_g_): (**a**) random, (**b**) attractive, and (**c**) repulsive distribution. In the present case, the mixing ratio *x* is 1/6. So, the probability to find *M*_g_ at the nearest-neighboring site is 1/6 in (**a**) random distribution, while it is higher (lower) than 1/6 in the (**b**) attractive [(**c**) repulsive] distribution.

The partial substitution of the host transition metal (*M*_h_) to the guest transition metal (*M*_g_) is an effective method to improve the functionality of the materials, such as its electrochemical^1–6^, magnetic^7–9^, and dielectric^10^ properties. For example, NaFe_0.5_Co_0.5_O_2_^1^ with an O3-type layered structure shows a high discharge capacity of 160 mAh/g and good cyclability, which is much higher than those of the parent O3-NaFeO_2_ and O3-NaCoO_2_. To microscopically comprehend the substitution effect, degree of distribution (random, attractive, and repulsive distribution: Fig. 1) of *M*_g_ is a crucial parameter. In the random distribution (Fig. 1a), the probability to find *M*_g_ at the nearest-neighboring site of *M*_g_ is the same as the mixing ratio (*x*) of *M*_g_. The probability is higher than *x* in the attractive distribution (Fig. 1b) while it is lower than *x* in the repulsive distribution (Fig. 1c). Thus, we can describe the degree of distribution with parameter *s* that modifies the probability to find *M*_g_ at the nearest-neighboring site as *sx*. *s* = 1, > 1, < 1 represent the random, attractive, and repulsive distribution, respectively. Here, we propose that a systematic extended X-ray absorption fine structure (EXAFS) analysis against the *M*_g_ concentration can reveal the spatial distribution of *M*_g_. The K-edge EXAFS analysis of 3*d* transition metals (*M*s) is a powerful technique to determine the local intermetal distance around the corresponding *M* in the mixed compounds. Importantly, the observed intermetal distance [*d*_*M*g*–M*_(*x*)] around *M*_g_ is the probability-weighted average of *d*_*M*g*–M*g_ and *d*_*M*g*–M*h_, because a difference between *d*_*M*g*–M*g_ and *d*_*M*g*–M*h_ is too small to separate. Then, we can extract the parameter *s* from systematic *d*_*M*g*–M*_(*x*) data against *x*.

The O3-type layered transition metal oxides, O3-Na*M*O_2_ (*M* = Cr, Fe, and Co), show quite simple crystal structure with alternating *M*O_2_ layers and Na sheets^11^. The *M*O_2_ layer consists of edge-sharing *M*O_6_ octahedra formed by covalent bonding. Especially, O3-NaFeO_2_ and O3-NaCoO_2_ form solid solution in the entire composition range. O3-NaFeO_2_ has been investigated due to its electrochemical^12^, magnetic^13,14^ properties. On the other hand, O3-NaCoO_2_ has been investigated due to its electrochemical^15^, thermoelectric^16^, and superconductive^17^ properties. In addition, the ionic radius (*r* = 0.645 Å) of high-spin Fe^3+^ is much larger than that (*r* = 0.545 Å) of low-spin Co^3+^. In this sense, O3-NaCo_1−*x*_Fe_*x*_O_2_ is a suitable system for investigation of degree of distribution of Fe.

We chose NaCo_1−*x*_Fe_*x*_O_2_ as a prototypical transition metal compound and systematically investigated the local intermetal distance around the host Co [*d*_Co–*M*_(*x*)] and the guest Fe [*d*_Fe–*M*_(*x*)] against *x*. The *x*-dependence of *d*_Fe–*M*_(*x*) was analyzed by an empirical equation, ${d}_{\mathrm{F}\mathrm{e}-M}(x)=sxd_{\mathrm{F}\mathrm{e}-\mathrm{F}\mathrm{e}}+(1-sx)d_{\mathrm{F}\mathrm{e}-\mathrm{C}\mathrm{o}}$, where *d*_Fe–Fe_ and *d*_Fe–Co_ are the Fe–Fe and Co–Fe distances, respectively. The obtained *s* value (= 4.8) indicates aggregation tendency of guest Fe in NaCo_1−*x*_Fe_*x*_O_2_.

## Results

### Local structure around transition metal

**Figure 2:**
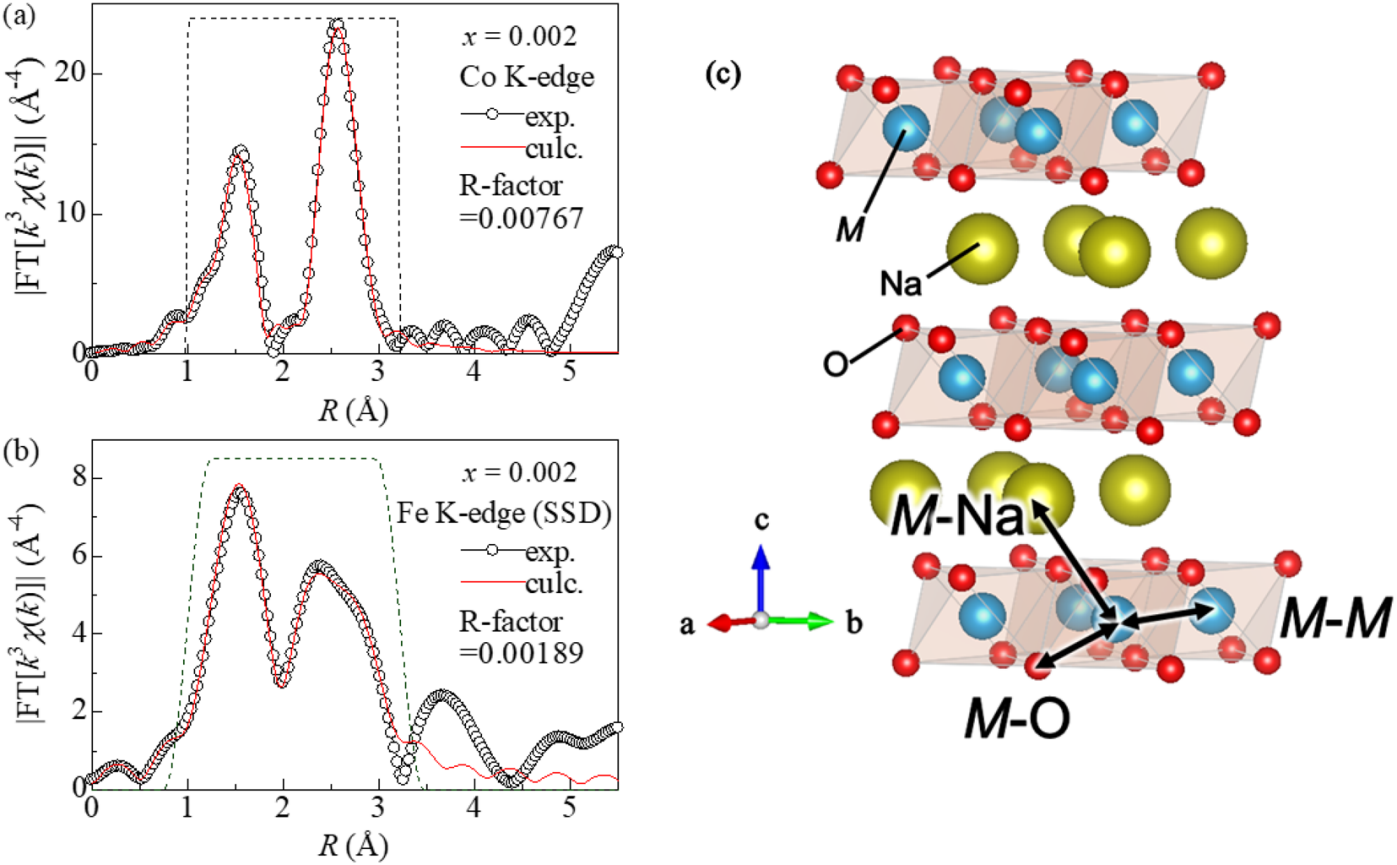
Fourier transforms of *k*^3^-weighted (**a**) Co and (**b**) Fe K-edge EXAFS spectra for NaCo_0.998_Fe_0.002_ without phase shift correction. Red curves are the results of the least-squares curve-fitting with the EXAFS equation in the *R* range from 1.0 to 3.22 Å. (**c**) Schematic structure of O3-Na*M*O_2_. The first- (*M*–O), the second- (*M*–*M*), and the third (*M*–Na) scattering paths are indicated by allows.

Figure 2a,b show prototypical Fourier transformation of the *χ*(*k*)*k*^3^–*R* plots around the host Co and the guest Fe in NaCo_0.998_Fe_0.002_O_2_, respectively. *χ* and *k* are the oscillatory components of the normalized absorption and wavenumber, respectively. In the Co K-edge EXAFS spectra (Fig. 2a), two intense peaks are observed at around 1.5 Å and 2.4 Å (without phase shift correction), which are assigned to the paths to the first- (O) and second- (*M*) nearest neighbor elements, respectively (Fig. 2c). The corresponding peaks are observed in the Fe K-edge EXAFS spectra (Fig. 2b). With including contributions from first-(O), second-(*M*), and third-(Na) nearest neighbor elements, we performed least-squares fittings with the EXAFS equation in the *R* range from 1.0 to 3.22 Å. Thus, obtained parameters around Co and Fe are listed in Tables 1 and 2, respectively. The Co–O distance [*d*_Co–O_ = 1.918(11) Å] and Co–Co distance [*d*_Co–Co_ = 2.875(9) Å] in NaCoO_2_ are close to the crystallographic values, 1.935 Å and 2.890 Å^18^, within experimental error.

**Table 1:** Structural parameters of NaCo_1−*x*_Fe_*x*_O_2_ obtained by Co K-edge EXAFS analyses.

*x*	*d*_Co–O_ (Å)	*σ*^2^_Co–O_ (Å^2^)	*d*_Co–*M*_ (Å)	*σ*^2^_Co–*M*_ (Å^2^)	*d*_Co–Na_ (Å)	*σ*^2^_Co–Na_ (Å^2^)
0.000	1.918 (11)	0.004 (1)	2.875 (9)	0.004 (1)	3.092 (10)	0.002 (2)
0.002	1.914 (10)	0.004 (1)	2.869 (8)	0.004 (1)	3.095 (10)	0.002 (1)
0.006	1.917 (10)	0.004 (1)	2.877 (8)	0.004 (1)	3.089 (9)	0.003 (2)
0.012	1.912 (7)	0.004 (1)	2.867 (6)	0.004 (1)	3.086 (8)	0.004 (1)
0.024	1.917 (9)	0.004 (1)	2.872 (6)	0.004 (1)	3.081 (10)	0.005 (2)
0.060	1.918 (6)	0.004 (1)	2.875 (5)	0.004 (1)	3.081 (8)	0.005 (2)

The spectra were recorded in transmission mode. *d*_Co–O_ and *d*_Co–*M*_ are Co–O and Co–*M* distances, respectively. *σ*^2^_Co–O_ and *σ*^2^_Co–*M*_ are the Debye–Waller factor for each shell. Uncertainty of the last digit(s) is given in parentheses.

**Table 2:** Structural parameters of NaCo_1−*x*_Fe_*x*_O_2_ obtained by Fe K-edge EXAFS analyses.

*x*	*d*_Fe–O_ (Å)	*σ*^2^_Fe–O_ (Å^2^)	*d*_Fe–*M*_ (Å)	*σ*^2^_Fe–*M*_ (Å^2^)	*d*_Fe–Na_ (Å)	*σ*^2^_Fe–Na_ (Å^2^)
0.002	2.003 (13)	0.005 (1)	2.916 (9)	0.006 (1)	3.116 (26)	0.013 (7)
0.006	1.995 (10)	0.007 (1)	2.915 (6)	0.007 (1)	3.120 (16)	0.012 (5)
0.012 (F)	1.995 (15)	0.009 (2)	2.910 (11)	0.008 (2)	3.100 (21)	0.009 (5)
0.012 (T)	2.003 (18)	0.005 (1)	2.919 (9)	0.004 (1)	3.169 (28)	0.011 (12)
0.024	2.004 (12)	0.003 (1)	2.923 (7)	0.005 (1)	3.160 (24)	0.013 (9)
0.060	2.014 (8)	0.003 (1)	2.947 (5)	0.006 (1)	3.153 (18)	0.016 (5)

The spectra were recorded in fluorescence (*x* = 0.002, 0.006, and 0.012 (F)) or transmission (*x* = 0.012 (T), 0.024, and 0.060) modes. *d*_Fe–O_ and *d*_Fe–*M*_ are Fe–O and Fe–*M* distances, respectively. *σ*^2^_Fe–O_ and *σ*^2^_Fe–*M*_ are the Debye–Waller factor for each shell. Uncertainty of the last digit(s) is given in parentheses.

Co and Fe K-edge X-ray absorption near edge structure (XANES) spectra for NaCo_1-*x*_Fe_*x*_O_2_ are shown in Figs. S1 and S2 in the supplementary information, respectively. We observed no detectable peak-shift in Co K-edge XANES spectra. We observed no detectable main peak shift for Fe K-edge XANES spectra, even though the spectral shape is slightly distorted due to the self-absorption effect^19^. These observations indicate that electronic states of the host Co and guest Fe are almost the same.

### Interatomic distances (*d*_Co–O_ and* d*_Fe–O_) to the nearest-neighboring oxygen

**Figure 3:**
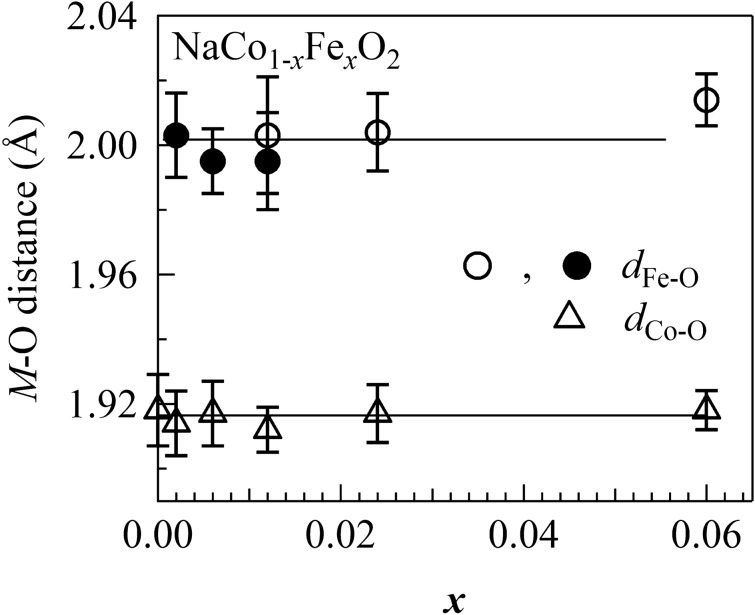
Co–O (*d*_Co–O_) and Fe–O (*d*_Fe–O_) distance of NaCo_1−*x*_Fe_*x*_O_2_ against *x*. Straight lines are guides for the eye. Open and closed symbols represent the corresponding EXAFS data obtained in the transmission and fluorescence modes, respectively.

Figure 3 shows the Co–O (*d*_Co–O_) and the Fe–O (*d*_Fe–O_) distances in NaCo_1−*x*_Fe_*x*_O_2_ against *x*. As indicated by the eye-guide straight lines, *d*_Co–O_ and *d*_Fe–O_ are almost constant against *x* within experimental error. The larger error bars in *d*_Fe–O_ are originated in the lower concentration of Fe and the resultant worse S/N ratio of the EXAFS signal. The robust nature of *d*_Co–O_ and *d*_Fe–O_ is ascribed to the fact that the Co (Fe) sites are surrounded by six oxygens as *M*O_6_ even in the mixed crystal. A similar robust nature of the interatomic distance to the first nearest elements is reported in the mixed crystal of metal-hexacyanides^20^, in which *M* is surrounded by six cyanide as *M*(NC)_6_.

### Interatomic distances (d_Co–M_ and d_Fe–M_) to the nearest-neighboring transition metals

**Figure 4:**
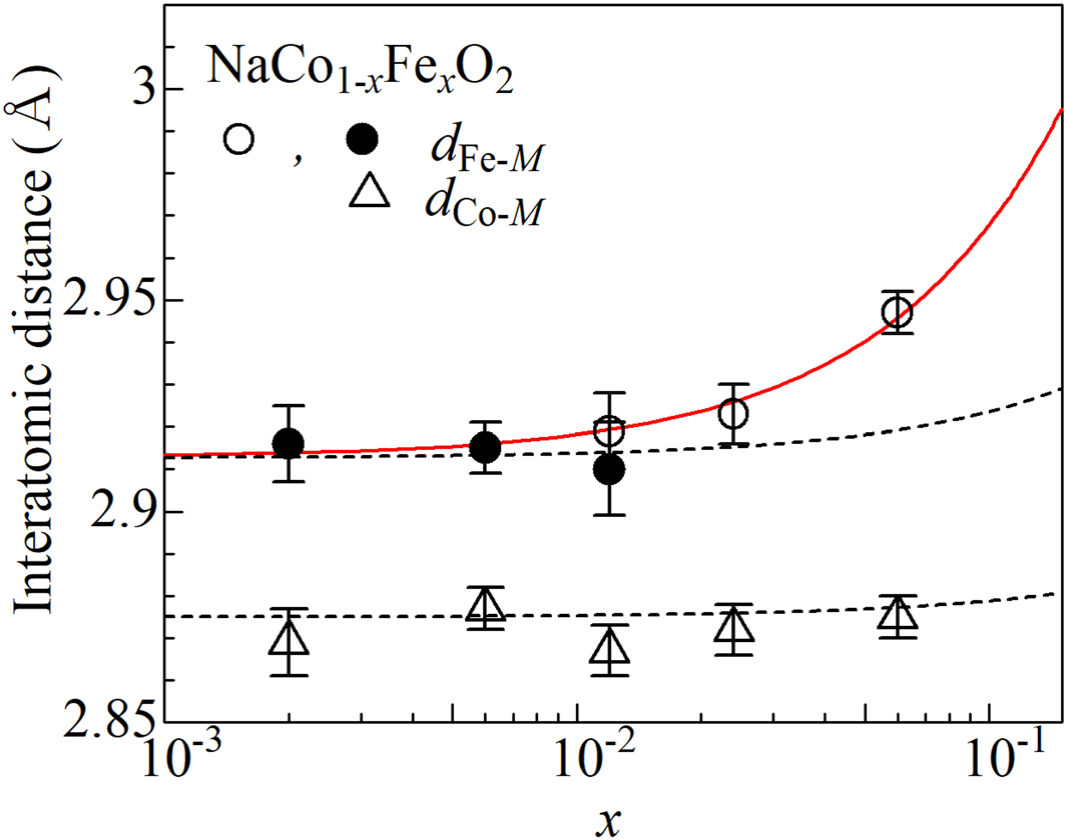
Co–*M* (*d*_Co–*M*_) and Fe–*M* (*d*_Fe–*M*_) distances of NaCo_1−*x*_Fe_*x*_O_2_ against *x*. Open and closed symbols represent the corresponding EXAFS data obtained in the transmission and fluorescence modes, respectively. Broken curves are calculated by ${d}_{\mathrm{C}\mathrm{o}-M}(x)=x{d}_{\mathrm{C}\mathrm{o}-\mathrm{F}\mathrm{e}}+(1-x){d}_{\mathrm{C}\mathrm{o}-\mathrm{C}\mathrm{o}}$ and ${d}_{\mathrm{F}\mathrm{e}-M}(x)=x{d}_{\mathrm{F}\mathrm{e}-\mathrm{F}\mathrm{e}}+(1-x){d}_{\mathrm{F}\mathrm{e}-\mathrm{C}\mathrm{o}}$, respectively. Red solid curve is the least-square fitting by the empirical equation; ${d}_{\mathrm{F}\mathrm{e}-M}(x)=sxd_{\mathrm{F}\mathrm{e}-\mathrm{F}\mathrm{e}}+(1-sx)d_{\mathrm{F}\mathrm{e}-\mathrm{C}\mathrm{o}}$ (*s* = 4.8).

Figure 4 shows the Fe–*M* (*d*_Fe–*M*_) and Co–*M* (*d*_Co–*M*_) distances in NaCo_1−*x*_Fe_*x*_O_2_ against *x*. *d*_Co–O_ is apparently robust against *x*. This is partly because difference between *d*_Co–Co_ [= 2.875(9) Å] and *d*_Co–Fe_ [= 2.91 Å; extrapolated value of *d*_Fe–*M*_(*x*) at *x* = 0] is too small to detect *x*-dependence of *d*_Co–*M*_(*x*). Looking at Fig. 4, one may notice that *d*_Fe–M_(*x*) shows significant *x*-dependence and increases with *x*, reflecting larger ionic radius of high-spin Fe^3+^ than that of low-spin Co^3+^.

First, let us consider the situation of random distribution of the guest Fe. As explained in the introduction section, *d*_Co–*M*_(*x*) [*d*_Fe–*M*_(*x*)] determined by the EXAFS analyses corresponds to the probability-weighted average of *d*_Co–Co_ and *d*_Co–Fe_ (*d*_Fe–Co_ and *d*_Fe–Fe_). In the random distribution of Fe, the probability to find Fe (Co) at the nearest-neighboring transition metal site is *x* (1 − *x*). Then, *d*_Co–*M*_(*x*) [*d*_Fe–*M*_(*x*)] is expressed as ${d}_{\mathrm{C}\mathrm{o}-M}(x)=x{d}_{\mathrm{C}\mathrm{o}-\mathrm{F}\mathrm{e}}+(1-x){d}_{\mathrm{C}\mathrm{o}-\mathrm{C}\mathrm{o}}$ [${d}_{\mathrm{F}\mathrm{e}-M}(x)=x{d}_{\mathrm{F}\mathrm{e}-\mathrm{F}\mathrm{e}}+(1-x){d}_{\mathrm{F}\mathrm{e}-\mathrm{C}\mathrm{o}}$], where *d*_Co–Co_ (= 2.875(9) Å; Table 1), *d*_Co–Fe_ (= *d*_Fe–Co_), and *d*_Fe–Fe_ are Co–Co, Co–Fe, and Fe–Fe distances, respectively. We use the crystallographic value (= 3.022 Å^18^) of NaFeO_2_ for *d*_Fe–Fe_ while we used the extrapolated value (= 2.913 Å) of *d*_Fe–*M*_ at *x* = 0 for *d*_Fe–Co_ (see Fig. 4). The broken curves in Fig. 4 is the calculation of *d*_Co–*M*_ (*x*) [*d*_Fe–*M*_(*x*)]. Concerning to *d*_Co–*M*_(*x*), the calculation under random distribution satisfactory reproduces the experimental data. Concerning to *d*_Fe–*M*_(*x*), the calculation fails to reproduce the experimental data. The deviation between experiment and calculation indicates deviation from the random distribution of Fe. Here, we modify the probability to find *M*_g_ at the nearest-neighboring site as *sx*. *s* = 1, > 1, < 1 represent the random, attractive, and repulsive distribution, respectively (Fig. 1). Then, we obtained empirical equation as

1 $${d}_{\mathrm{F}\mathrm{e}-M}\left(x\right)=sxd_{\mathrm{F}\mathrm{e}-\mathrm{F}\mathrm{e}}+\left(1-sx\right)d_{\mathrm{F}\mathrm{e}-\mathrm{C}\mathrm{o}}.$$

In general, an additional term such as constant*x(1 − *x*) is adopted to compensate the deviation between experimental data and the linear relation, because the term becomes zero at *x* = 0 and 1. We, however, applied a more straightforward expression (Eq. 1). The parameter (*s*) directly modifies the probability to find Fe at the nearest-neighboring site and has clear physical meaning. The red curve in Fig. 4 is the least-squares fitted results with Eq. (1). The fitted curve well reproduces the experimental data. The obtained *s* (= 4.8) is larger than unity, indicating that the Fe distribution has an aggregation tendency.

## Discussion

The origin of the aggregation tendency of Fe is probably the distortion energy originated in the difference in the ionic radius between high-spin Fe^3+^ (*r* = 0.645 Å) than low-spin Co^3+^ (*r* = 0.545 Å). Actually, the lattice constant (*a* = 3.022 Å, *c* = 16.074 Å^18^) of NaFeO_2_ is much larger than those (*a* = 2.890 Å, *c* = 15.609 Å^18^) of NaCoO_2_. Then, the region where Fe and Co are mixed has extra distortion energy. Reflecting to this extra distortion energy, NaCo_1−*x*_Fe_*x*_O_2_ has the phase-separation instability, or, spatially decomposition into (1 − *x*)NaCoO_2_ and *x*NaFeO_2_. In the free energy point of view, the phase separation significantly decreases the entropy, and hence, is not realized unless the gain of the internal energy is huge enough. Even if the phase separation is not realized, there remains the phase separation tendency, that is, the aggregation tendency of the guest Fe, as observed in Fig. 4.

In addition, we point out the Kinetics effect in synthesis. NaCo_1−*x*_Fe_*x*_O_2_ were synthesized by sloid state reaction at relatively lower temperature (~ 850 K). In this situation, atomic migrations within the compound is not enough to reach the ground state. As a result, the compound remains in the metastable state with relatively random distribution. Amaha et al.^21^ investigated chemical and structural inhomogeneity of two sets of NaFe_1/2_Co_1/2_O_2_: one was prepared by quenching and the other was prepared by slowly-cooling after the synthesis at 1,173 K, respectively. They observed traces of inhomogeneity in the slowly-cooled compound while no trace of inhomogeneity was observed in the quenched one. This observation is consistent with the aggregation tendency of the guest Fe as detected the systematic EXAFS analyses against *x* in the present study.

## Summary

We systematically investigated *d*_Co–*M*_(*x*) and *d*_Fe–*M*_(*x*) of NaCo_1−*x*_Fe_*x*_O_2_ against *x*. We found that *d*_Fe–*M*_(*x*) steeply increases with increases in *x*. The *x*-dependence of *d*_Fe–*M*_(*x*) was analyzed by an empirical equation, ${d}_{\mathrm{F}\mathrm{e}-M}(x)=sxd_{\mathrm{F}\mathrm{e}-\mathrm{F}\mathrm{e}}+(1-sx)d_{\mathrm{F}\mathrm{e}-\mathrm{C}\mathrm{o}}$. The obtained *s* value (= 4.8) indicates the aggregation tendency of the guest Fe in NaCo_1−*x*_Fe_*x*_O_2_. Thus, systematic EXAFS analysis against *M*_g_ concentration is a highly sensitive method to detect deviation from the random distribution of *M*_g_ in partially substituted materials.

## Methods

### Sample preparation and characterization

Layered oxides NaCo_1−*x*_Fe_*x*_O_2_ were prepared by solid state reaction. Na_2_O_2_, Co_3_O_4_, and Fe_2_O_3_ were mixed in a 1.2:1–*n*:*n* atomic ratio and calcined at 858 K for 20 h in O_2_. *n* (= 0, 0.002, 0.005, 0.010, 0.020, and 0.005) is the nominal Fe content. Then, the product was finely ground, and again calcined in the same condition. The actual Fe content (*x*) in NaCo_1−*x*_Fe_*x*_O_2_ were determined by the inductively-coupled plasma (ICP) method as shown in Fig. S3 (*x* = 0, 0.002, 0.006, 0.012, 0.024, 0.060, respectively).

The X-ray diffraction (XRD) patterns were obtained using an X-ray powder diffractometer (MultiFlex, Rigaku, Tokyo, Japan) with the Bragg–Brentano (*θ*–2*θ*) geometry. The X-ray source was the Cu Kα line (λ = 1.54 Å) operated at 40 kV and 40 mA. The observed diffraction peaks can be indexed with O3-type structure (*R* $\stackrel{-}{3}$ m; *Z* = 3) without detectable impurities such as defect-spinel phases (Fig. S4). The lattice constants *a* and *c* were refined by the Rietveld analysis (Rietan-FP^22^) with a trigonal model (*R* $\stackrel{-}{3}$ m; *Z* = 3, hexagonal setting). Reflecting the larger ionic radius of Fe^3+^, *a* and *c* increase with *x* (Fig. S5).

### Local structural analysis by EXAFS

The extended X-ray absorption fine structure (EXAFS) measurements were conducted at BL-9A beamline of the Photon Factory, KEK. The synchrotron radiation was monochromatized by a Si (111) double-crystal monochromator. The energy resolution (ΔE/E) was ~ 2 × 10^−4^ and the photon flux at sample position was ~ 4 × 10^11^ phs/s. The samples were finely ground, mixed with BN, and pressed into pellets with 5 mm in diameter. Co K-edge EXAFS spectra of NaCo_1−*x*_Fe_*x*_O_2_ with *x* = 0.000, 0.002, 0.006, 0.012, 0.024, and 0.060 and Fe K-edge EXAFS spectra of NaCo_1−*x*_Fe_*x*_O_2_ with *x* = 0.012, 0.024, and 0.060 were recorded in transmission mode with a gas-filled ion chamber at room temperature. Fe K-edge EXAFS spectra of NaCo_1−*x*_Fe_*x*_O_2_ with *x* = 0.002, 0.006, and 0.012 were recorded in fluorescence mode using a 19-element solid-state detector (SSD). Before the SSD, a Mn filleter was used to remove the background signal from Co Kα fluorescence.

The background subtraction, normalization and analyses of EXAFS spectra were performed using the ATHENA program and EXAFS analyses were performed using the ARTEMIS programs^23^ as described elsewhere^18,20^. First, the oscillatory components were extracted using the ATHENA program after background subtraction and normalization of the absorption spectra. Thus, we obtained *χ*(*k*)*k*^3^ – *k* plots, where *χ* and *k* are the oscillatory components of the normalized absorption and angular wavenumber, respectively. Co and Fe K-edge EXAFS oscillations without any modelling results for NaCo_1-*x*_Fe_*x*_O_2_ are shown in Figs. S6 and S7, respectively. The Fourier transformations of the χ(*k*)*k*^3^–R plots were performed in the *k*-range from 3.0 to 14.0 Å^−1^ at Co K-edge and from 2.0 to 8.5 Å^−1^ at Fe K-edge using the ATHENA program. In the plane wave and single-scattering approximation, χ(*k*) around the K-edge is expressed by the EXAFS equation as:

2 $$\upchi \left(k\right)=-{S}_{0}^{2}\sum_{j}\frac{{N}_{j}}{k{R}_{j}^{2}}{F}_{j}\left(k\right){e}^{-2{\sigma }_{j}{k}^{2}}\sin\left\{2k{R}_{j}+{\phi }_{j}\left(k\right)\right\},$$

where *S*_0_, *N*_j_, *R*_j_, *F*_j_, *σ*_j_^2^, and *φ*_j_ are the passive electron reduction factor, degeneracy of path, path length, effective scattering amplitude, mean square displacement, and effective scattering phase shift of the *j*th atom, respectively. The *k* is defined by $k=\sqrt{2m(E-{E}_{o})/\hslash }$ where *m*, *E*, and *E*_0_ are the electron mass, energy of the incident X-ray, and energy shift, respectively. In the least-squares curve fitting, we included the contribution from first (O), second (TM), and third (Na) nearest neighbor elements. We fixed *N*_j_ for the three elements at the crystallographic value (*N*_1_ = *N*_2_ = *N*_3_ = 6). We used the same *S*_0_ parameter for the three elements. The least-squares fittings were performed in *q*-space, which is inverse Fourier transformation in the *R* range from 1 to 3.22 Å. The best fitted results are shown in Figs. S8–S14.

Here, we consider the validity of the number of the free parameters (*N*_fp_) in the EXAFS analysis. In general, *N*_fp_ should not exceed the maximum number of independent parameters (*N*_idp_ = 2*ΔkΔR*/π)^24^. For Co K-edge EXAFS, *N*_idp_ is 15 since *Δk* is 11 Å^−1^ and *ΔR* is 2.22 Å. For Fe K-edge EXAFS, *N*_idp_ is 9 since *Δk* is 6.5 Å^−1^ and *ΔR* is 2.22 Å. Even if we exclude the *k*-range less than 3 which is not relevant to the EXAFS, *N*_idp_ for Fe K-edge EXAFS is 8. The free parameters used for analysis are *S*_0_^2^, *E*_0_, *d*_Fe–O_, *d*_Fe–M_, *d*_Fe–Na_, *σ*_Fe–O_, *σ*_Fe–M_, *σ*_Fe–Na_ for Fe K-edge EXAFS spectrum. Since *S*_0_^2^ is common for six spectra, the substantial *N*_fp_ is 7 + 1/6 for each Fe K-edge EXAFS spectrum. Similarly, the free parameters used for analysis are *S*_0_^2^, *E*_0_, *d*_Co–O_, *d*_Co–M_, *d*_Co–Na_, *σ*_Co–O_, *σ*_Co–M_, *σ*_Co–Na_ for Co K-edge EXAFS spectrum. The substantial *N*_fp_ is 7 + 1/6 for each Co K-edge EXAFS spectrum because *S*_0_^2^ is common for six Co spectra. Therefore. *N*_fp_ (= 7 + 1/6) is less than *N*_idp_ for both the Co and Fe K-edge EXAFS spectra.

Here, let us consider the difference between the error bar (σ_*r*_) of interatomic distance (*r*) obtained by the EXAFS analysis and the experimental resolution (*Δr*) in Fourier transformed *R*-space. σ_*r*_ (e.g. 0.005–0.011 Å for *d*_Fe-M_) is determined by the least-squares fitting of the experimental data by EXAFS Eq. (2). In general, the χ^2^ statistic is defined as^25^

3 $${\chi }^{2}\equiv \sum_{i=1}^{n}\left\{\frac{1}{{\sigma }_{i}^{2}}{\left[{y}_{i}-y({x}_{i})\right]}^{2}\right\}$$

where *y*_*i*_, *y*(*x*_*i*_), and σ_*i*_ are the observed values, calculated values, and uncertainty in *y*_*i*_, respectively. In the present case, *y*(*x*_*i*_) are evaluated by the EXAFS Eq. (2) at *x*_*i*_. Then, σ_*r*_ is given by.

4 $${\sigma }_{r}=\sqrt{2{\left(\frac{{\partial }^{2}{\chi }^{2}}{\partial {r}^{2}}\right)}^{-1}}.$$

On the other hand, Δ*r* is the lower limit value at which two peaks (shells) are separated and is given by *Δr* = π/2*Δk*, where *Δk* = *k*_max_ − *k*_min_^26^. *Δr* for Fe (Co) K-edge EXAFS spectrum is 0.24 (0.14) Å. Thus, *Δr* is different from σ_*r*._

## Supplementary information

Supplementary file1 (PDF 1551 kb)
