# Supplementary material for: Aggregation tendency of guest Fe in NaCo1−xFexO2 (x < 0.1) as investigated by systematic EXAFS analysis
Source: Sci Rep. 2020 Jul 9;10:11283. doi: 10.1038/s41598-020-68147-3 (PMC7347642; doi:10.1038/s41598-020-68147-3)
Supplement: Supplementary file 1 — Supplementary file1 (PDF 1551 kb) [file 41598_2020_68147_MOESM1_ESM.pdf]

Supplementary information

Aggregation tendency of guest Fe in $NaCo_{1-x}Fe_xO_2$ (x < 0.1) as investigated by systematic EXAFS analysis

Toshiaki Moriya[1], Hideharu Niwa[1-3]*, Hiroaki Nitani[4], Yutaka Moritomo[1-3]*

[1]*Graduate School of Pure and Applied Sciences, University of Tsukuba, Tsukuba 305-8571, Japan*

[2]*Faculty of Pure and Applied Sciences, University of Tsukuba, Tsukuba 305-8571, Japan*

[3]*Tsukuba Research Center for Energy Materials Science (TREMS), University of Tsukuba, Tsukuba 305-8571, Japan*

[4]*Institute of Materials Science, High Energy Accelerator Research Organization (KEK), Tsukuba 305-0801, Japan*

E-mail: niwa.hideharu.ga@u.tsukuba.ac.jp, moritomo.yutaka.gf@u.tsukuba.ac.jp

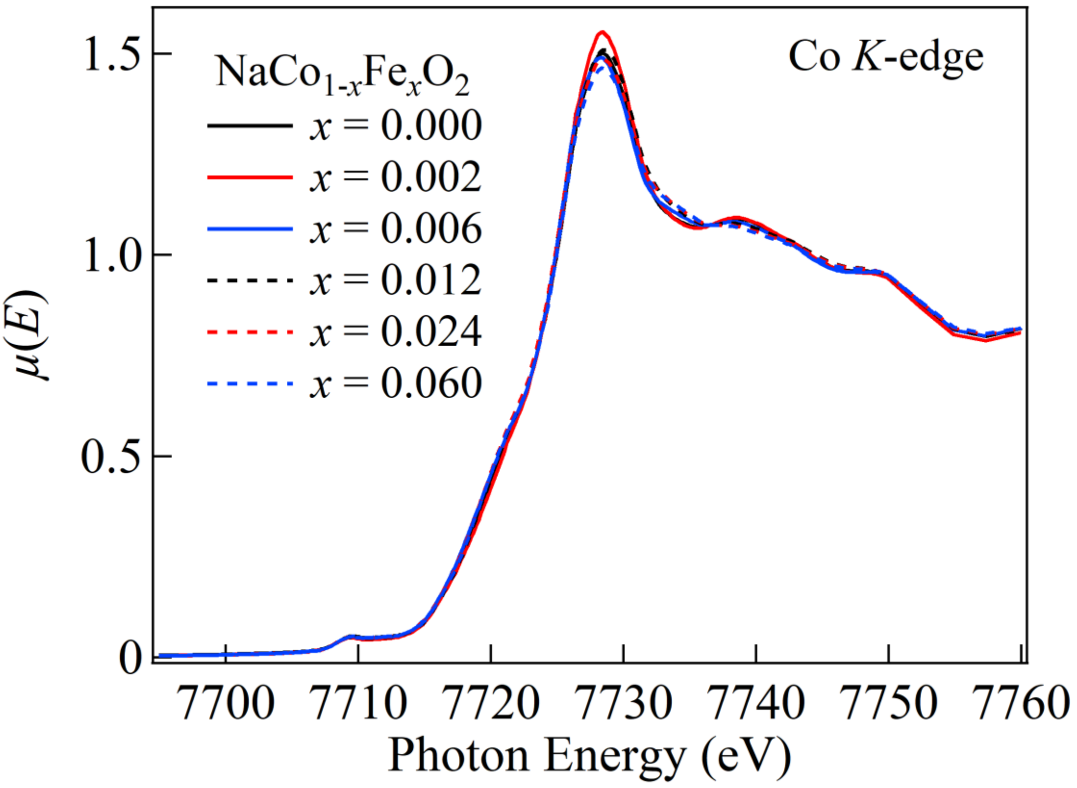

Figure S1. Co K-edge XANES spectra for $NaCo_{1-x}Fe_xO_2$.

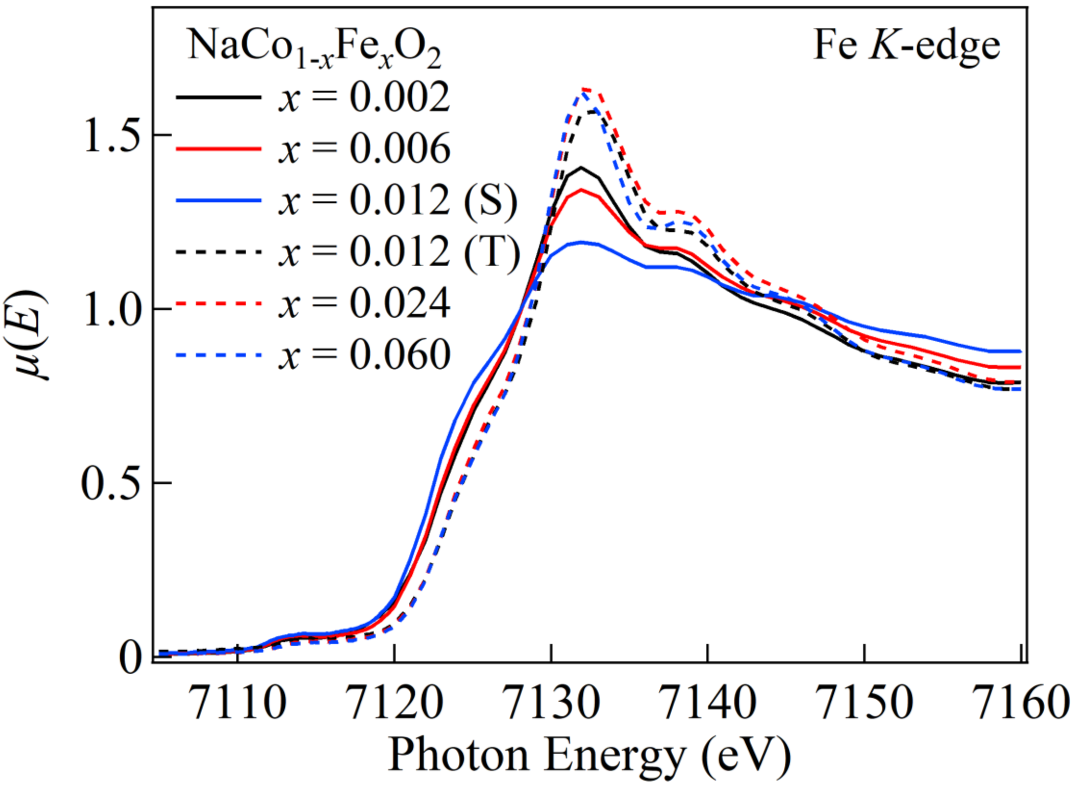

Figure S2. Fe K-edge XANES spectra for $NaCo_{1-x}Fe_xO_2$. The spectra were recorded in fluorescence (x = 0.002, 0.006, and 0.012 (F)) or transmission (x = 0.012 (T), 0.024, and 0.060) modes.

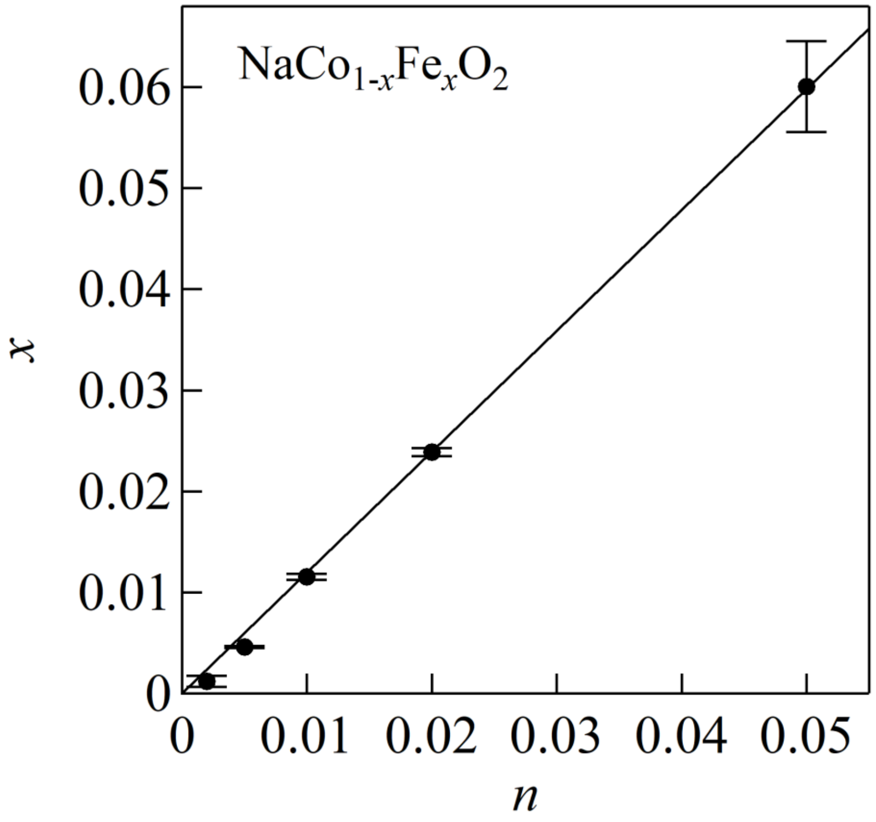

Figure S3. Fe content x in $NaCo_{1-x}Fe_xO_2$ determined by ICP measurements versus nominal Fe content (n). Errors are standard deviation of two measurements. The solid line indicates the result of the least-squares fitting: $x = 1.196n$.

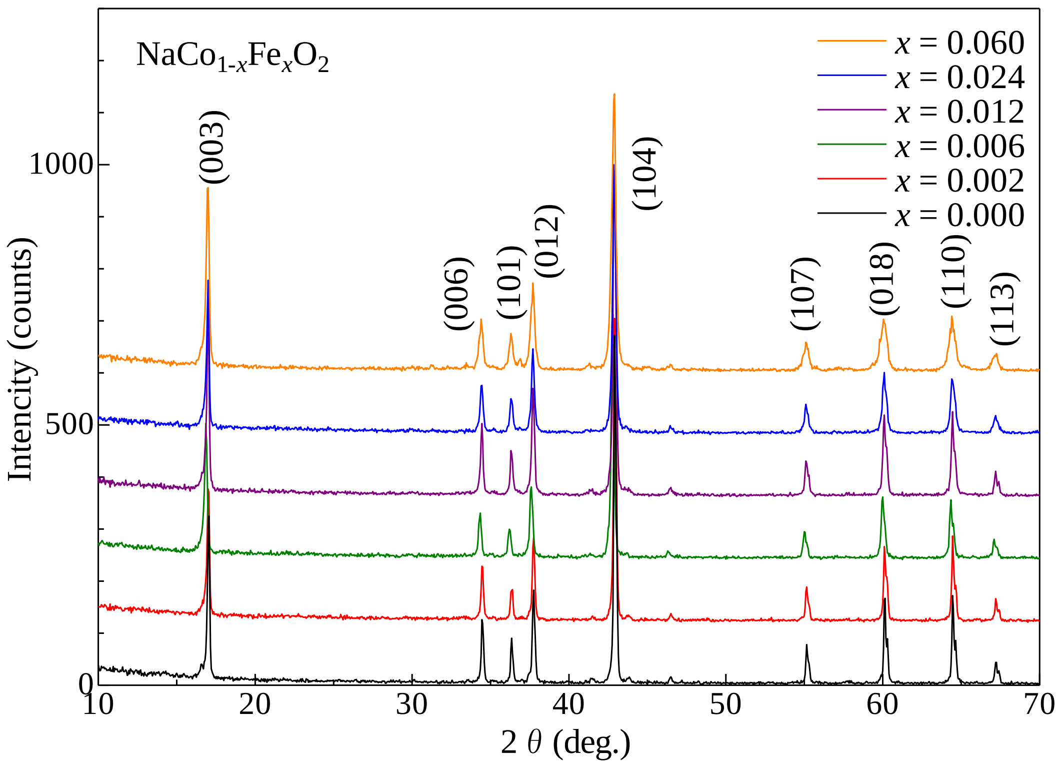

Figure S4. XRD patterns of $NaCo_{1-x}Fe_xO_2$ against x. X-ray source was Cu K_α line. Values in parentheses represent indexes in the trigonal structure.

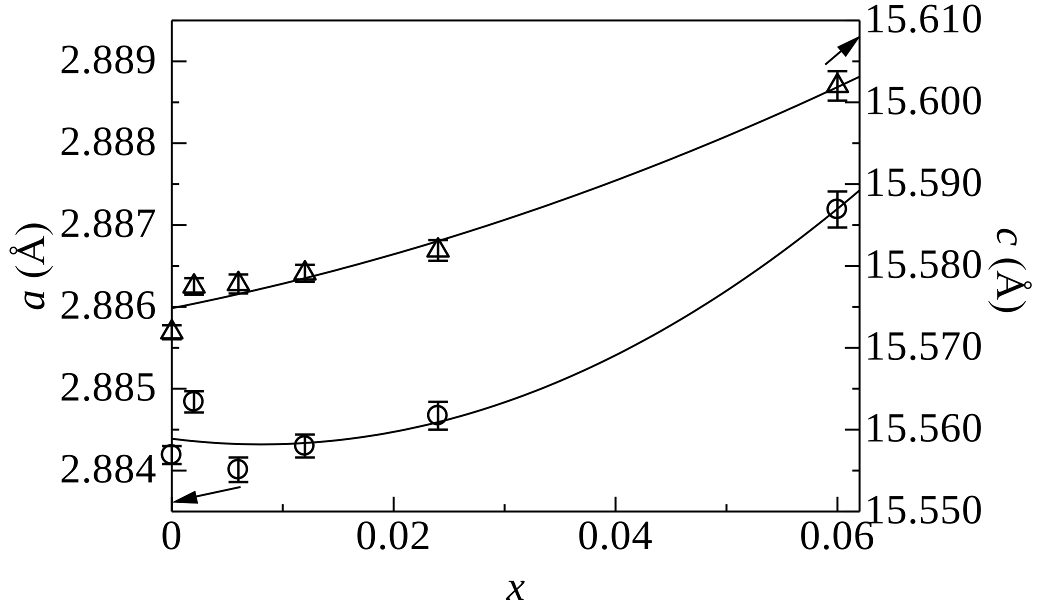

Figure S5. Lattice constants *a* (circle) and *c* (triangle) of $NaCo_{1-x}Fe_xO_2$ against *x*. Solid curves are eye-guided lines.

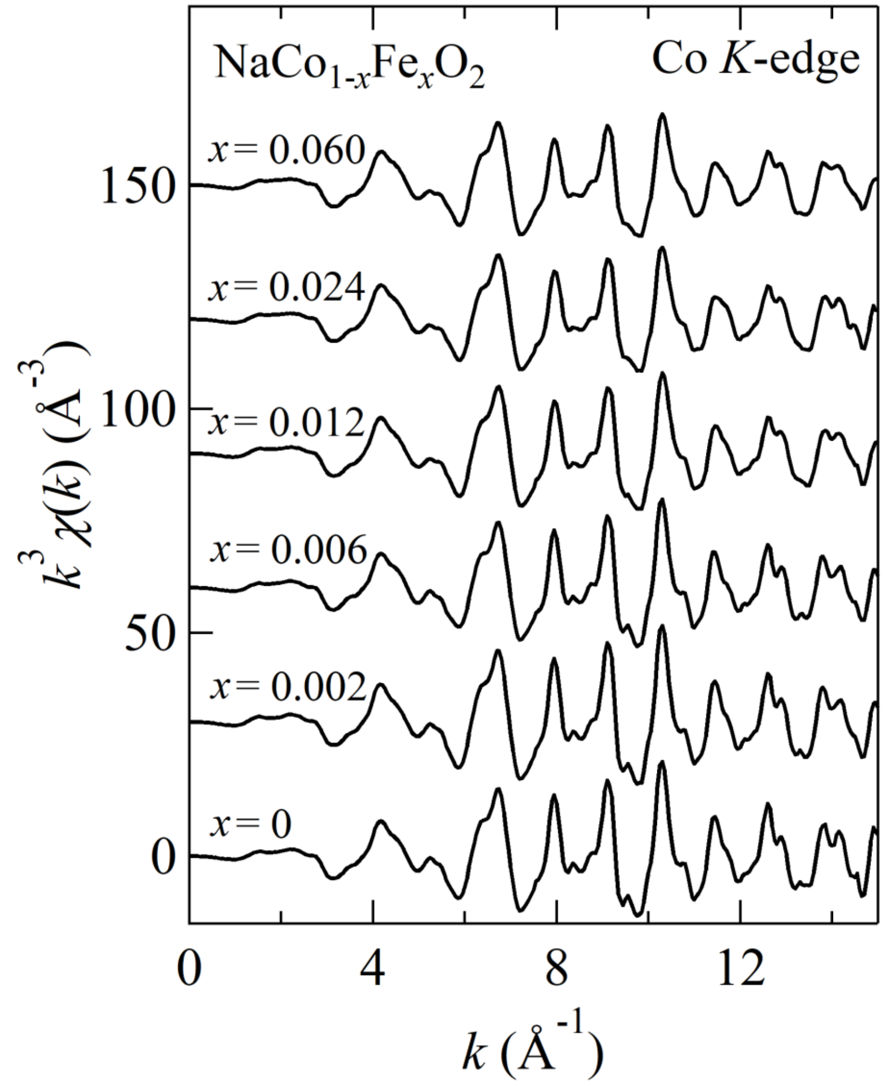

Figure S6. Co K-edge EXAFS oscillations for $NaCo_{1-x}Fe_xO_2$.

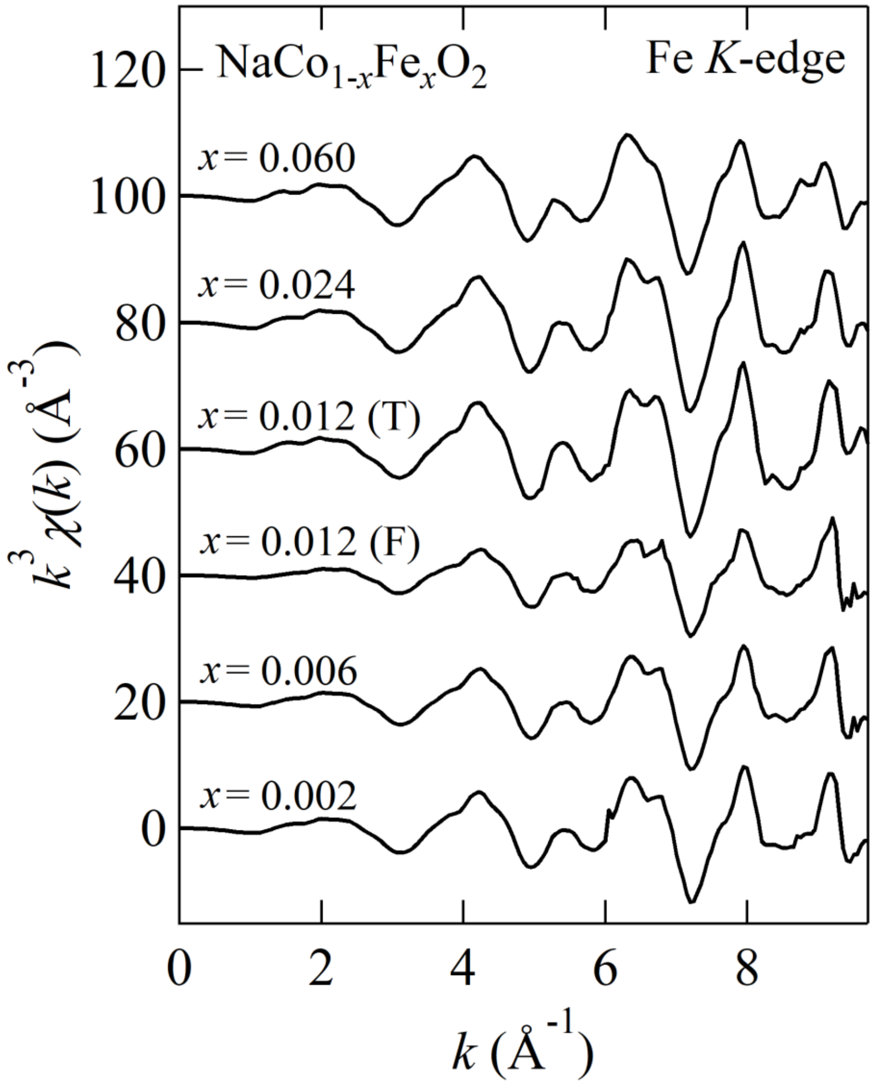

Figure S7. Fe K-edge EXAFS oscillations for $NaCo_{1-x}Fe_xO_2$. The spectra were recorded in fluorescence (x = 0.002, 0.006, and 0.012 (F)) or transmission (x = 0.012 (T), 0.024, and 0.060) modes.

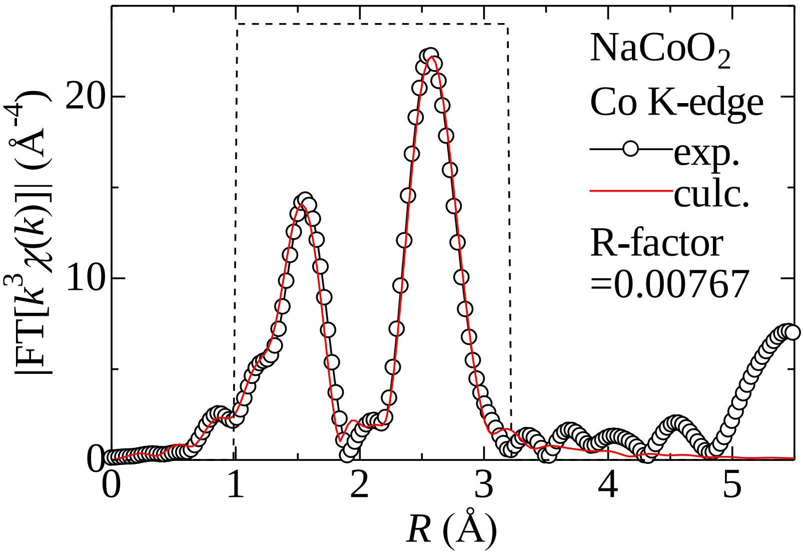

Figure S8. FT[$\chi(k)k^3$]−R plots at Co K-edge for $NaCoO_2$. Red curve is the least-squares fitting with the EXAFS equation in the R range from 1 Å to 3.22 Å. The degeneracy of path N_j is fixed to 6. The obtained parameters are the following. S_0^2, E_0, R_j, σ_j^2, are the passive electron reduction factor, threshold energy, path length, and mean square displacement (DW factor), respectively.

Path	S_0^2	N_j	E_0 (eV)	R_j (Å)	σ_j^2 (Å^2)
Co–O	0.82(5)	6	7717(2)	1.918(11)	0.004(1)
Co–*M*		6		2.875(9)	0.004(1)
Co–Na		6		3.092(10)	0.002(2)

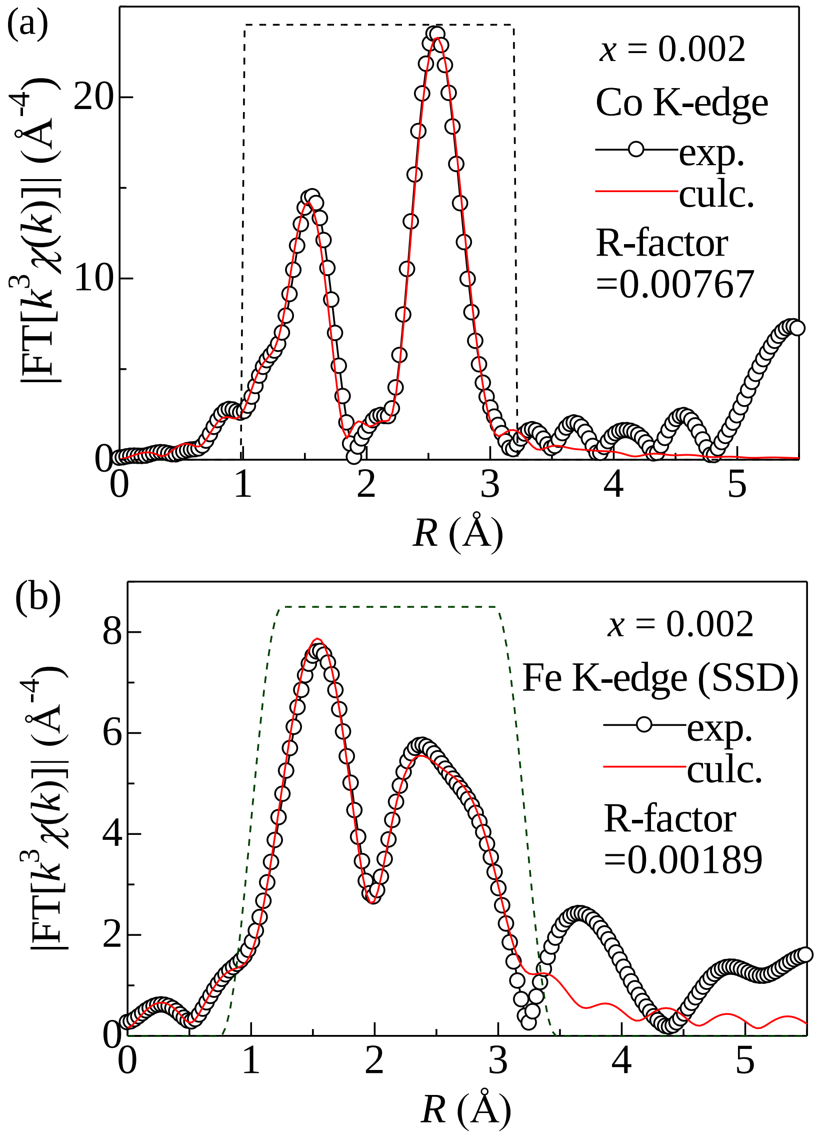

Figure S9. FT[$\chi(k)k^3$]−R plots at (a) Co and (b) Fe K-edges for $NaCo_{0.998}Fe_{0.002}O_2$. Fe K-edge spectrum was recorded with fluorescence mode. Red curve is the least-squares fitting with the EXAFS equation in the R range from 1 Å to 3.22 Å. The parameters are the following.

Path	S_0^2	N_j	E_0 (eV)	R_j (Å)	σ_j^2 (Å^2)
Co–O	0.82(5)	6	7716(2)	1.914(10)	0.004(1)
Co–*M*		6		2.869(8)	0.004(1)
Co–Na		6		3.095(20)	0.002(1)
Fe–O	0.75(6)	6	7123(2)	2.003(13)	0.005(1)
Fe–*M*		6		2.916(9)	0.006(1)
Fe–Na		6		3.116(26)	0.013(7)

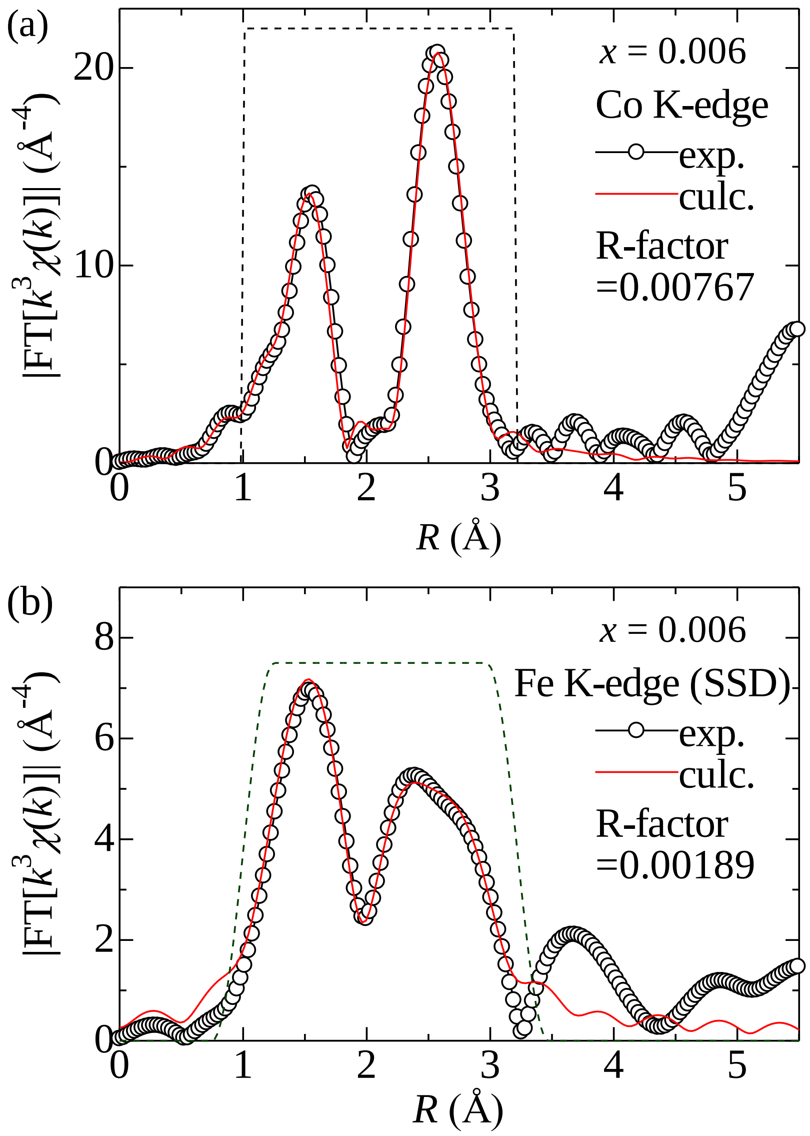

Figure S10. FT[$\chi(k)k^3$]−R plots at (a) Co and (b) Fe K-edges for $NaCo_{0.994}Fe_{0.006}O_2$. Fe K-edge spectrum was recorded with fluorescence mode.Red curve is the least-squares fitting with the EXAFS equation in the R range from 1 Å to 3.22 Å. The parameters are the following.

Path	S_0^2	N_j	E_0 (eV)	R_j (Å)	σ_j^2 (Å^2)
Co–O		6		1.917(10)	0.004(1)
Co–*M*	0.82(5)	6	7717(2)	2.877(8)	0.004(1)
Co–Na		6		3.089(9)	0.003(2)
Fe–O		6		1.995(10)	0.007(1)
Fe–*M*	0.75(6)	6	7123(2)	2.915(6)	0.007(1)
Fe–Na		6		3.120(16)	0.012(5)

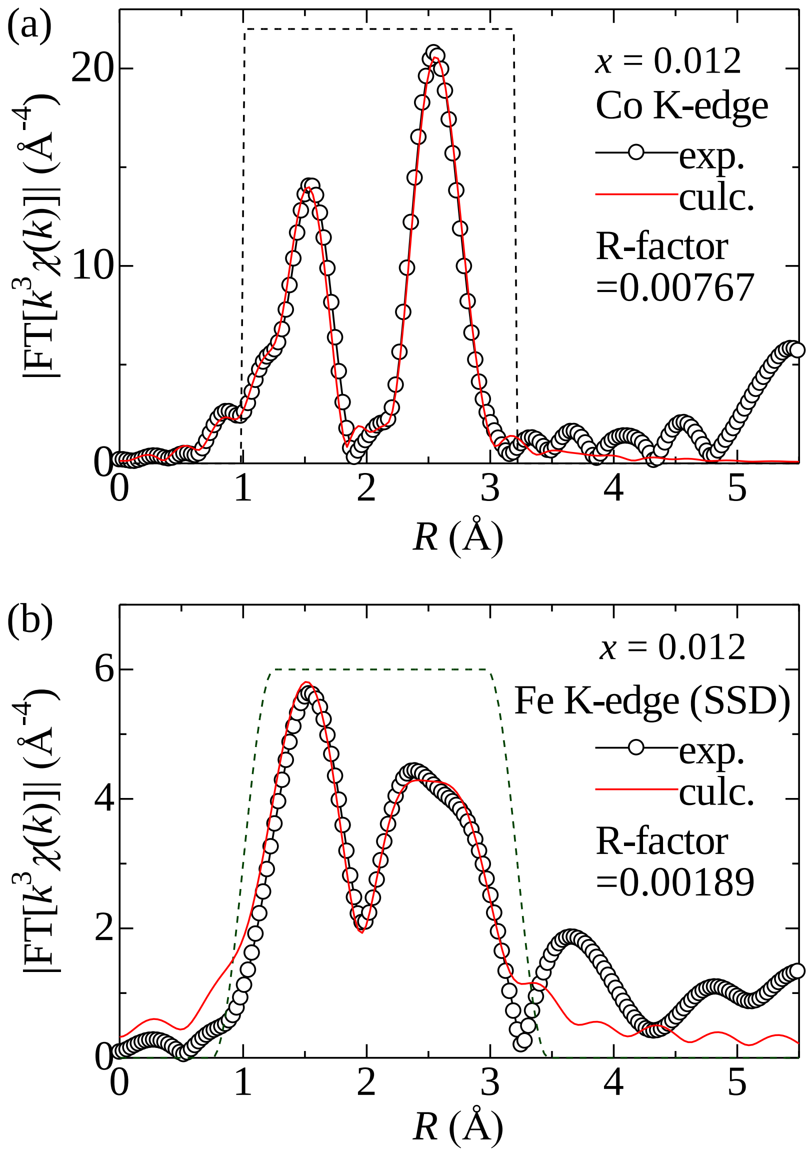

Figure S11. FT[$\chi(k)k^3$]−R plots at (a) Co and (b) Fe K-edges for $NaCo_{0.988}Fe_{0.012}O_2$. Fe K-edge spectrum was recorded with fluorescence mode. Red curve is the least-squares fitting with the EXAFS equation in the R range from 1 Å to 3.22 Å. The parameters are the following.

Path	S_0^2	N_j	E_0 (eV)	R_j (Å)	σ_j^2 (Å^2)
Co–O	0.82(5)	6	7717(2)	1.912(7)	0.004(1)
Co–M		6		2.867(6)	0.004(1)
Co–Na		6		3.086(8)	0.004(1)
Fe–O	0.75(6)	6	7123(2)	1.995(15)	0.009(2)
Fe–M		6		2.910(11)	0.008(2)
Fe–Na		6		3.100(21)	0.009(5)

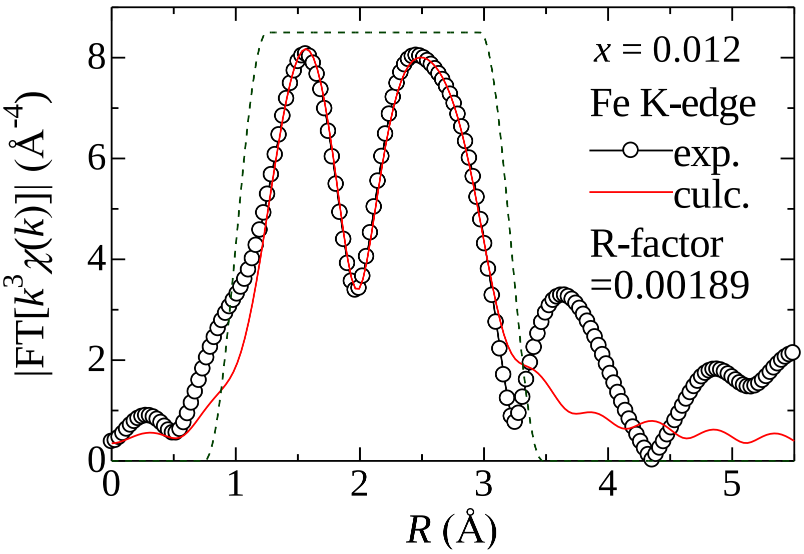

Figure S12. FT[$\chi(k)k^3$]−R plot at Fe K-edge for $NaCo_{0.988}Fe_{0.012}O_2$. Fe K-edge spectrum was recorded with transmission mode. Red curve is the least-squares fitting with the EXAFS equation in the R range from 1 Å to 3.22 Å. The parameters are the following.

Path	S_0^2	N_j	E_0 (eV)	R_j (Å)	σ_j^2 (Å^2)
Fe–O	0.75(6)	6	7126(3)	2.003(18)	0.005(1)
Fe–*M*		6		2.919(9)	0.004(1)
Fe–Na		6		3.169(28)	0.011(12)

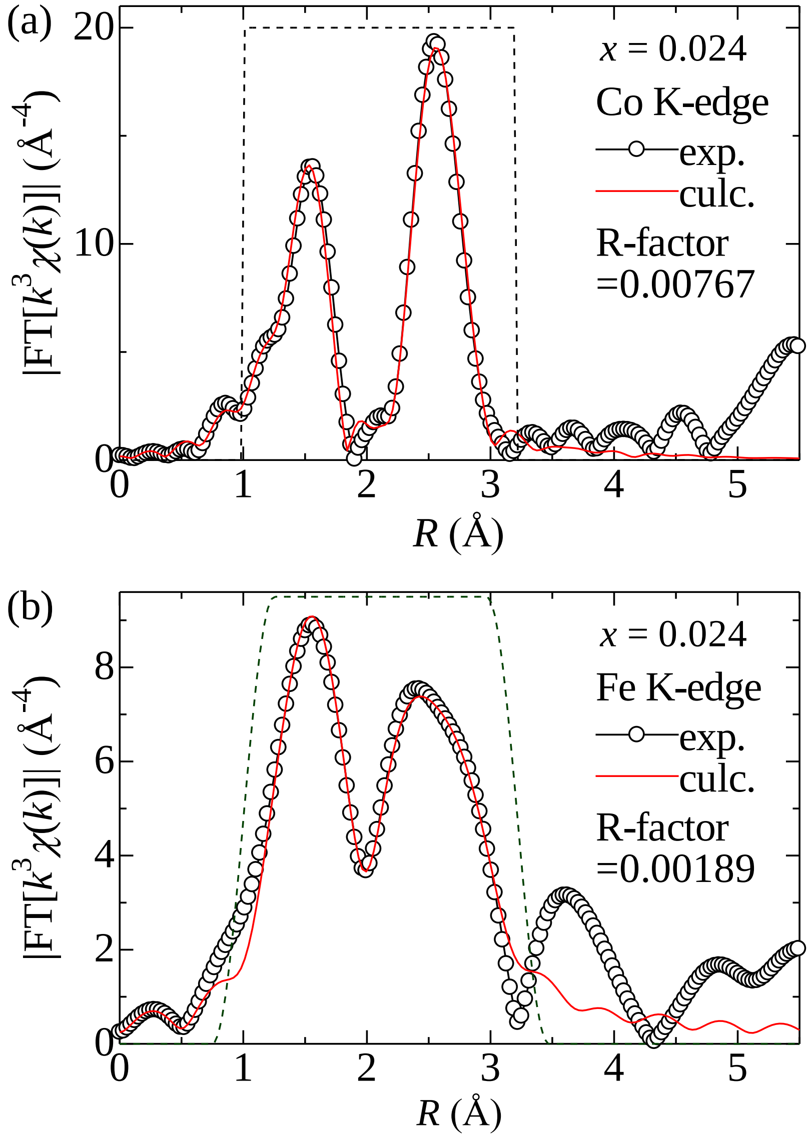

Figure S13. FT[$\chi(k)k^3$]$-R$ plot plots at (a) Co and (b) Fe K-edges for $NaCo_{0.976}Fe_{0.024}O_2$. Fe K-edge spectrum was recorded with transmission mode. Red curve is the least-squares fitting with the EXAFS equation in the *R* range from 1 Å to 3.22 Å. The parameters are the following.

Path	S_0^2	N_j	E_0 (eV)	R_j (Å)	σ_j^2 (Å^2)
Co–O		6		1.917(9)	0.004(1)
Co–*M*	0.82(5)	6	7718(2)	2.872(6)	0.004(1)
Co–Na		6		3.081(10)	0.005(2)
Fe–O		6		2.004(12)	0.003(1)
Fe–*M*	0.75(6)	6	7126(3)	2.923(7)	0.005(1)
Fe–Na		6		3.160(24)	0.013(9)

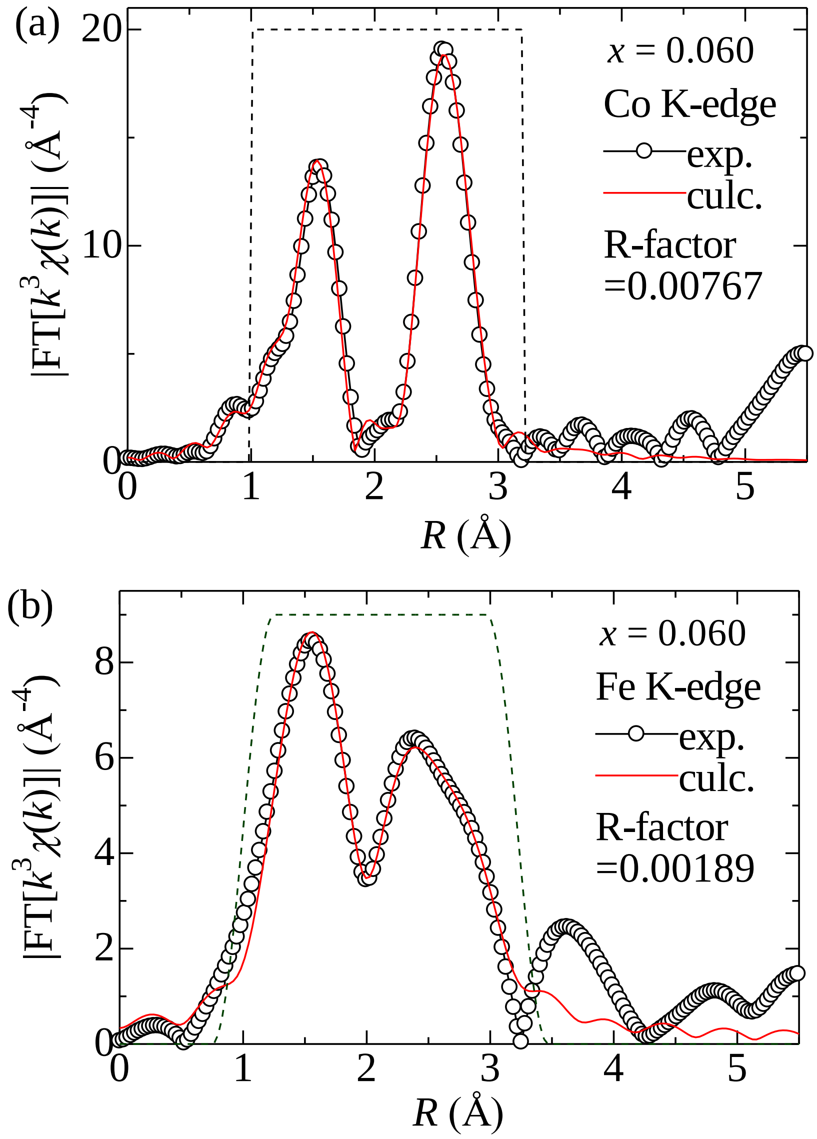

Figure S14. FT[$\chi(k)k^3$]−R plot plots at (a) Co and (b) Fe K-edges for $NaCo_{0.950}Fe_{0.050}O_2$. Fe K-edge spectrum was recorded with transmission mode. Red curve is the least-squares fitting with the EXAFS equation in the R range from 1 Å to 3.22 Å. The parameters are the following.

Path	S_0^2	N_j	E_0 (eV)	R_j (Å)	σ_j^2 (Å^2)
Co–O	0.82(5)	6	7718(2)	1.918(6)	0.004(1)
Co–*M*		6		2.875(5)	0.004(1)
Co–Na		6		3.081(8)	0.005(2)
Fe–O	0.75(6)	6	7126(3)	2.014(8)	0.003(1)
Fe–*M*		6		2.947(5)	0.006(1)
Fe–Na		6		3.153(18)	0.016(5)
